# Supplementary material for: The scaffolding function of LSD1 controls DNA methylation in mouse ESCs
Source: Nat Commun. 2024 Sep 5;15:7758. doi: 10.1038/s41467-024-51966-7 (PMC11377572; doi:10.1038/s41467-024-51966-7)
Supplement: Supplementary file 1 — Supplementary Information [file 41467_2024_51966_MOESM1_ESM.docx]

**The scaffolding function of LSD1 controls DNA methylation in mouse ESCs**

Sandhya Malla^1,2^, Kanchan Kumari^1,2^, Carlos A. García-Prieto^3,4^, Jonatan Caroli^5^, Anna Nordin^6,7^, Trinh T.T.Phan^8^, Devi Prasad Bhattarai^1,2^, Carlos Martinez-Gamero^1,2^, Eshagh Dorafshan^1,2^, Stephanie Stransky^9^, Damiana Álvarez-Errico^3^, Paulina Avovome Saiki^1,2^, Weiyi Lai^10^, Cong Lyu^10^, Ludvig Lizana^11^, Jonathan D. Gilthorpe^12^, Hailin Wang^10^, Simone Sidoli^9^, Andre Mateus^13,14^, Dung-Fang Lee^8,15,16,17^, Claudio Cantú^6,7^, Manel Esteller^3,18,19,20^, Andrea Mattevi^5^, Angel Roman^21^, Francesca Aguilo^1,2*^

**Supplementary Fig. 1:**

**
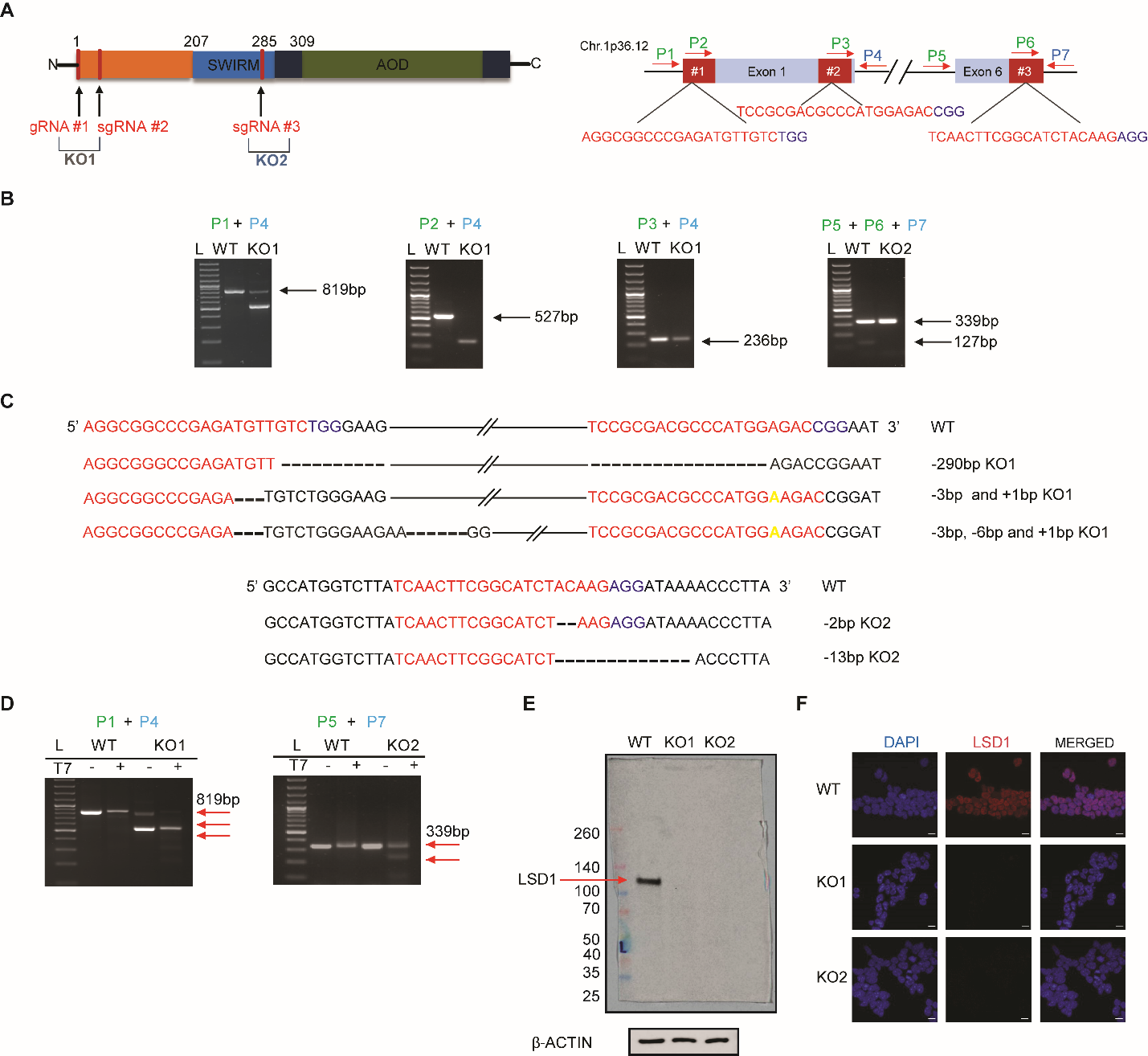
**

*Figure legends on the next page.*

**Supplementary Fig. 1: Generation of *Lsd1* KO mouse ESCs, related to Fig. 1**

(A) Schematic diagram of sgRNAs targeting the regions in the LSD1 protein (left panel) and the exons 1 and 6 of the *Lsd1* genomic sequence. The sgRNAs and PAM sequences are marked in red and blue, respectively. Only *Lsd1* isoform 1 is depicted for simplification.

(B) Agarose gel electrophoresis for validating *Lsd1* KO1 and KO2 mouse ESCs. PCR products were amplified using the primers P1-P7 (P1, P2, P3, P5, and P6: forward primers; P4 and P7: reverse primers) depicted in panel (A).

(C) Sanger sequencing analysis of *Lsd1* KO1 (top panel) and KO2 (bottom panel) shows insertions and deletions represented in yellow and dashes, respectively.

(D) T7 Endonuclease 1 assay of genomic DNA extracted from WT, *Lsd1* KO1, and *Lsd1* KO2 mouse ESCs. Heteroduplexes generated multiple bands after digestion, which are shown in the red arrow. The primers used for PCR amplification were P1 and P4 and P5 and P7.

(E) Representative full western blot of LSD1 on whole-cell extracts (WCE) from WT and *Lsd1* KO mouse ESCs. β-ACTIN is used as the loading control.

(F) Immunofluorescence analysis of LSD1 in WT and *Lsd1* KO mouse ESCs. DAPI was used as a nuclear marker.  Scale bar, 20 µm.

Results are one representative of n = 3 independent biological experiments (D, E and F). Uncropped blots are represented in the source data.

**Supplementary Fig. 2:**

**
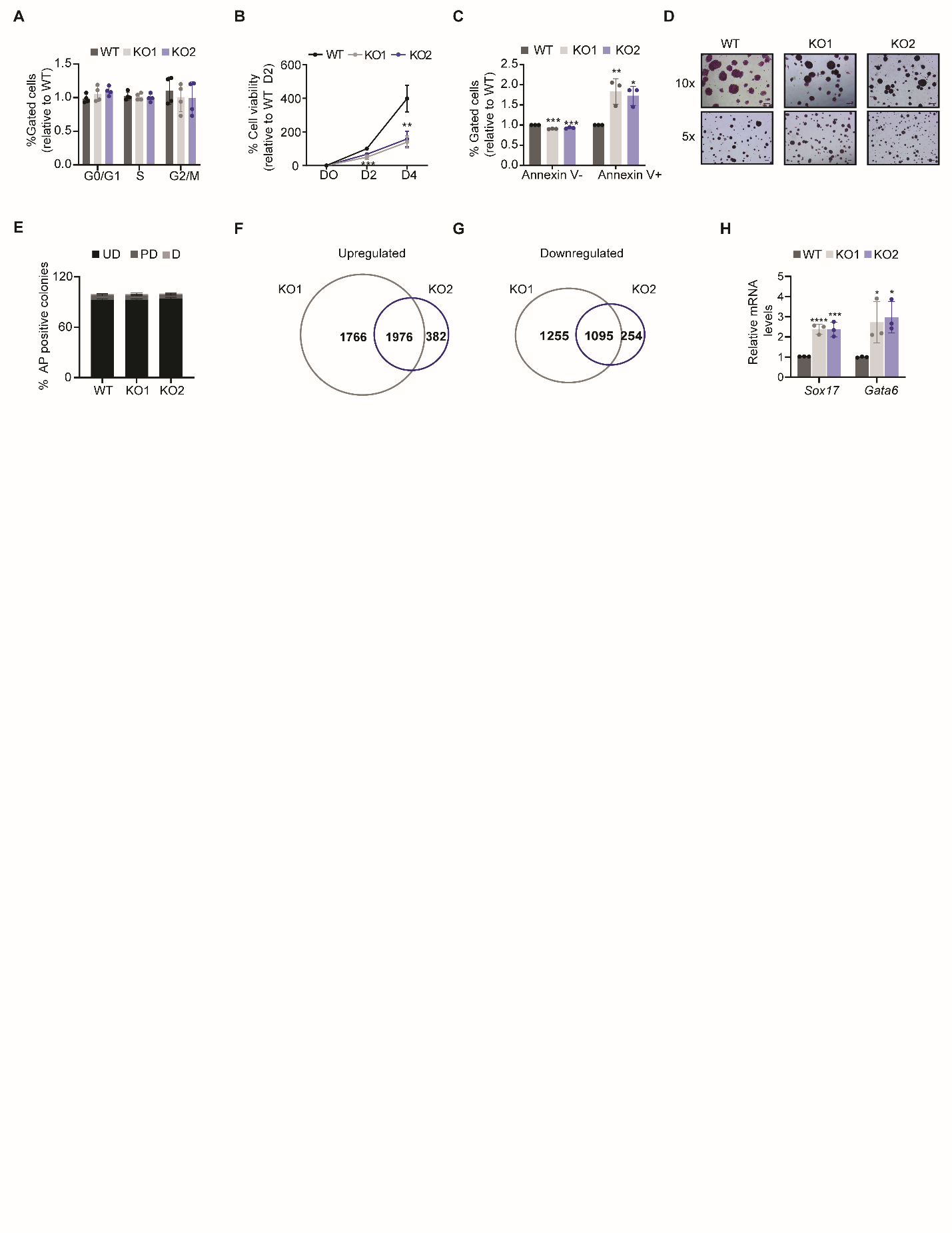
**

**Supplementary Fig. 2: LSD1 is dispensable for the maintenance of the naïve pluripotent state, related to Fig. 1**

(A) Cell cycle profile in *Lsd1* KO ESCs relative to WT mouse ESCs.

(B) Percentage of cell viability of WT and *Lsd1* KO mouse ESCs grown in 2iL medium.

(C) Percentage of live (Annexin V-) and apoptotic cells (Annexin V+) in *Lsd1* KO ESCs relative to WT mouse ESCs grown in 2iL medium.

(D and E) (D) AP staining images and (E) quantification of colonies in WT and *Lsd1* KO mouse ESCs grown in 2iL medium. Undifferentiated (UD), partially differentiated (PD), and differentiated (D). Scale bars, 50 μm.

(F and G) Venn diagram showing the overlap of the (F) upregulated and (G) downregulated genes between *Lsd1* KO mouse ESCs retrieved from RNA-seq (FC >1.5 and p < 0.05).

(H) RT-qPCR analysis of the endoderm markers (*Sox17* and *Gata6*) in WT and *Lsd1* KO mouse ESCs.

Statistical analysis: Two- tailed unpaired t-test (B, E, and H) and ordinary one-ANOVA (A and C). ∗p < 0.05, ∗∗p < 0.01, ∗∗∗p < 0.001, ∗∗∗∗p < 0.0001 and ns = non-significant (A and C). Error bars denote mean ± SD; n = (B = 4 and E = 3). Each dot in the bar graphs represents independent biological replicates (A, C and H). Results are one representative of n = 3 independent biological experiments (D).

**Supplementary Fig. 3:**


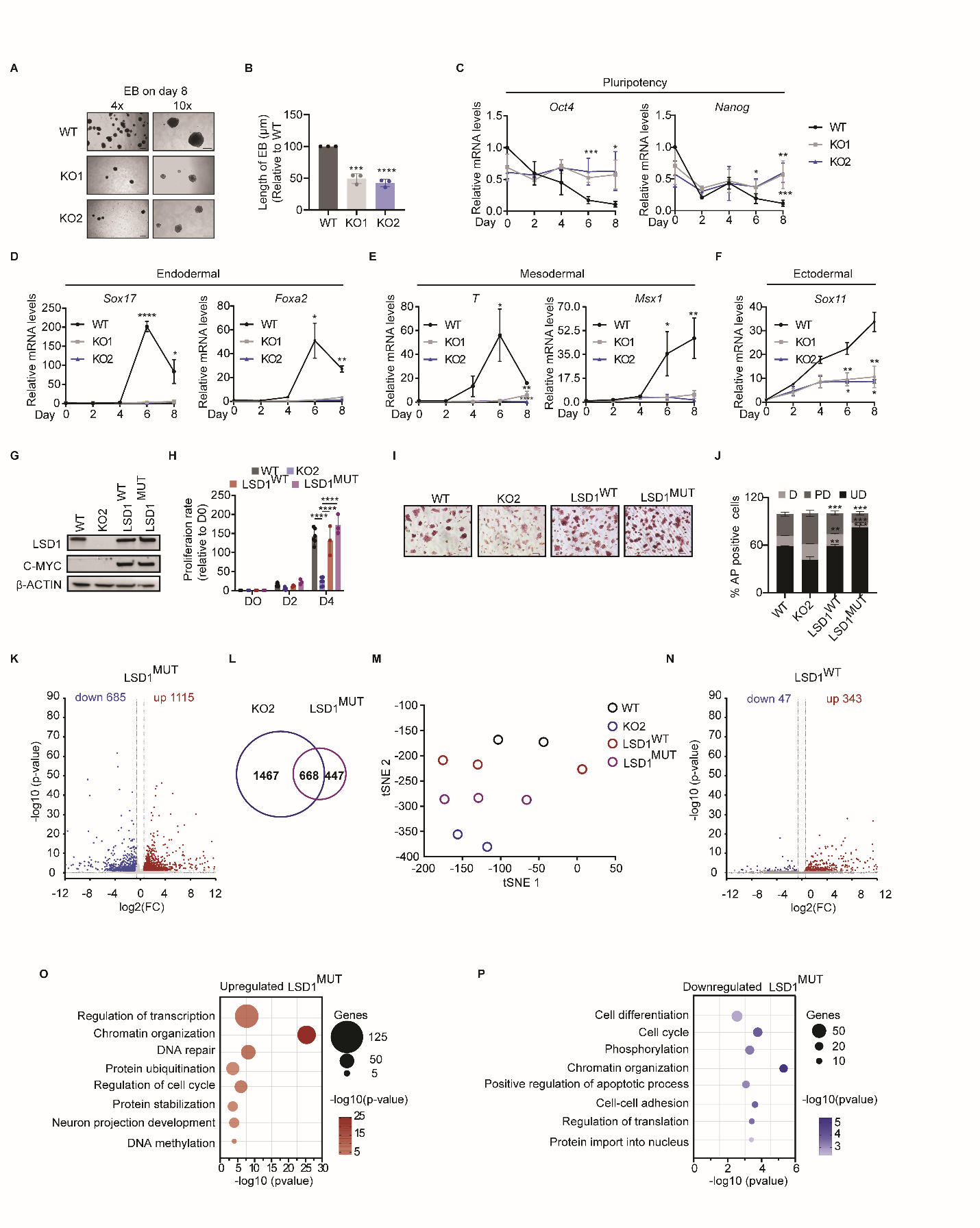


*Figure legends on the next page.*

**Supplementary Fig. 3: *Lsd1* deletion halts differentiation, related to Fig. 2**

(A and B) (A) Representative bright field images at (4x (left) and 10x (right)) magnification and (B) quantification of the size of EB derived from WT and *Lsd1* KO ESCs on the 8th day of differentiation. Scale bars, 200 µm.

(C-F) RT-qPCR analysis of (C) the pluripotency (*Oct4* and *Nanog*), (D) the endodermal (*Sox17* and *Foxa2*), (E) the mesodermal (*T* and *Msx1*), and (F) the ectodermal (*Sox11*) markers in WT, *Lsd1* KO1 and *Lsd1* KO2 mouse ESCs. mRNA levels are relative to the expression of WT at day 0.

1. Western blot of C-MYC and LSD1 on WCE of WT, *Lsd1* KO2, LSD1^WT^_,_ and LSD1^MUT^ mouse ESCs. β-ACTIN is used as the loading control.
2. Relative cell proliferation rate of WT, *Lsd1* KO2, LSD1^WT,^ and LSD1^MUT^ mouse ESCs assessed over 4 days.

(I and J) (I) AP staining images and (J) quantification of colonies in WT, *Lsd1* KO2, LSD1^WT,^ and LSD1^MUT^ mouse ESCs. Undifferentiated (UD), partially differentiated (PD), and differentiated (D). Scale bars, 50 μm.

(K) Volcano plots of differentially expressed transcripts in LSD1^MUT^ in comparison to WT mouse ESCs. Significant upregulated and downregulated transcripts are represented in red and blue, respectively (p < 0.05 and Fold change (FC) > 1.5). Non-significant hits are shown in grey dots.

(L) Venn diagram showing the overlap of all upregulated transcripts between *Lsd1* KO and LSD1^MUT^ mouse ESCs (FC >1.5 and p < 0.05).

(M) Principal component analysis of all transcripts in WT, *Lsd1* KO2, LSD1^WT,^ and LSD1^MUT^ mouse ESCs retrieved from RNA-sequencing.

(N) Volcano plots of differentially expressed transcripts in LSD1^WT^ in comparison to WT mouse ESCs. Significantly upregulated and downregulated transcripts are represented in red and blue, respectively (p < 0.05 and Fold change (FC) > 1.5). Non-significant hits are shown in grey dots. FDR value was calculated with the Benjamini–Hochberg correction.

(O and P) Gene ontology (GO) analysis of biological processes related to the (O) upregulated and (P) downregulated genes of LSD1^MUT^ ESCs compared to WT mouse ESCs (p < 0.05 and FC > 1.5). P-values were adjusted with the Benjamini–Hochberg correction.

Statistical analysis: Two-tailed unpaired t-test (B-F, and J) and ordinary one-way ANOVA (H). ∗p < 0.05, ∗∗∗p < 0.001, and ∗∗∗∗p < 0.0001. Error bars denote mean ± SD; n ≥ 3 (C-F expect Day 2 and Day 4 where n = 2); n = 3 (J (KO versus D1 WT, and LSD1^MUT^ ESCs)). Each dot in the bar graphs represents independent biological replicates (B and H). Results are one representative of n = 3 independent biological experiments (A, G, and I). Uncropped blots are represented in the source data.

**Supplementary Fig. 4:**

**
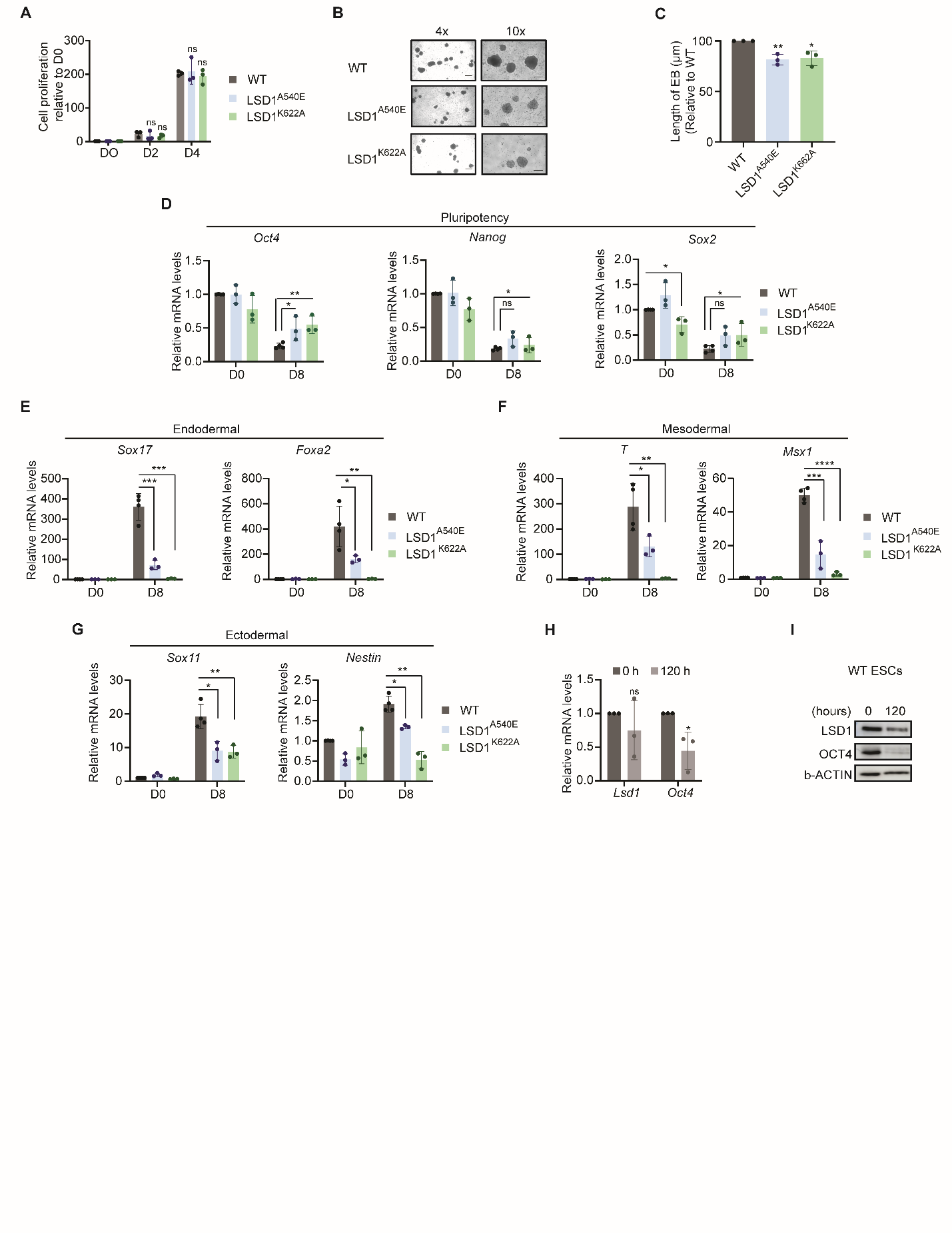
**

*Figure legends on the next page.*

**Supplementary Fig. 4: Mutation in LSD1 protein affects differentiation, related to Fig. 2**

1. Relative cell proliferation rate of WT, LSD1^A540E,^ and LSD1^K622A^ mouse ESCs assessed over 4 days.

(B and C) (B) Representative bright field images at (4x (left) and 10x (right)) magnification and (C) quantification of the size of EB derived from WT, LSD1^A540E,^ and LSD1^K622A^ mouse ESCs on the 8th day of differentiation. Scale bars, 200 µm.

(D-G) RT-qPCR analysis of (D) the pluripotency (*Oct4*, *Nanog and Sox2*), (E) the endodermal (*Sox17* and *Foxa2*), (F) the mesodermal (*T* and *Msx1*), and (G) the ectodermal (*Sox11 and Nestin*) markers in WT, LSD1^A540E,^ and LSD1^K622A^ mouse ESCs on the indicated time points. The mRNA levels are relative to the expression of WT at day 0.

(H and I) (H) RT-qPCR of *Lsd1* and *Oct4* and (I) Western blot of LSD1 and OCT4 on WCE of gastruloids generated from WT mouse ESCs on indicated time points. The mRNA levels are relative to the expression of expression of WT at 0 h.

Statistical analysis: Two-tailed unpaired t-test (A, C, D-G, and H). ∗p < 0.05, ∗∗∗p < 0.001, ∗∗∗∗p < 0.0001 and ns = non-significant. Error bars denote mean ± SD. Each dot in the bar graphs represents independent biological replicates (A, C, D-G and H). Results are one representative of n = 3 independent biological experiments (B and I). Uncropped blots are represented in the source data.

**Supplementary Fig. 5:**

**
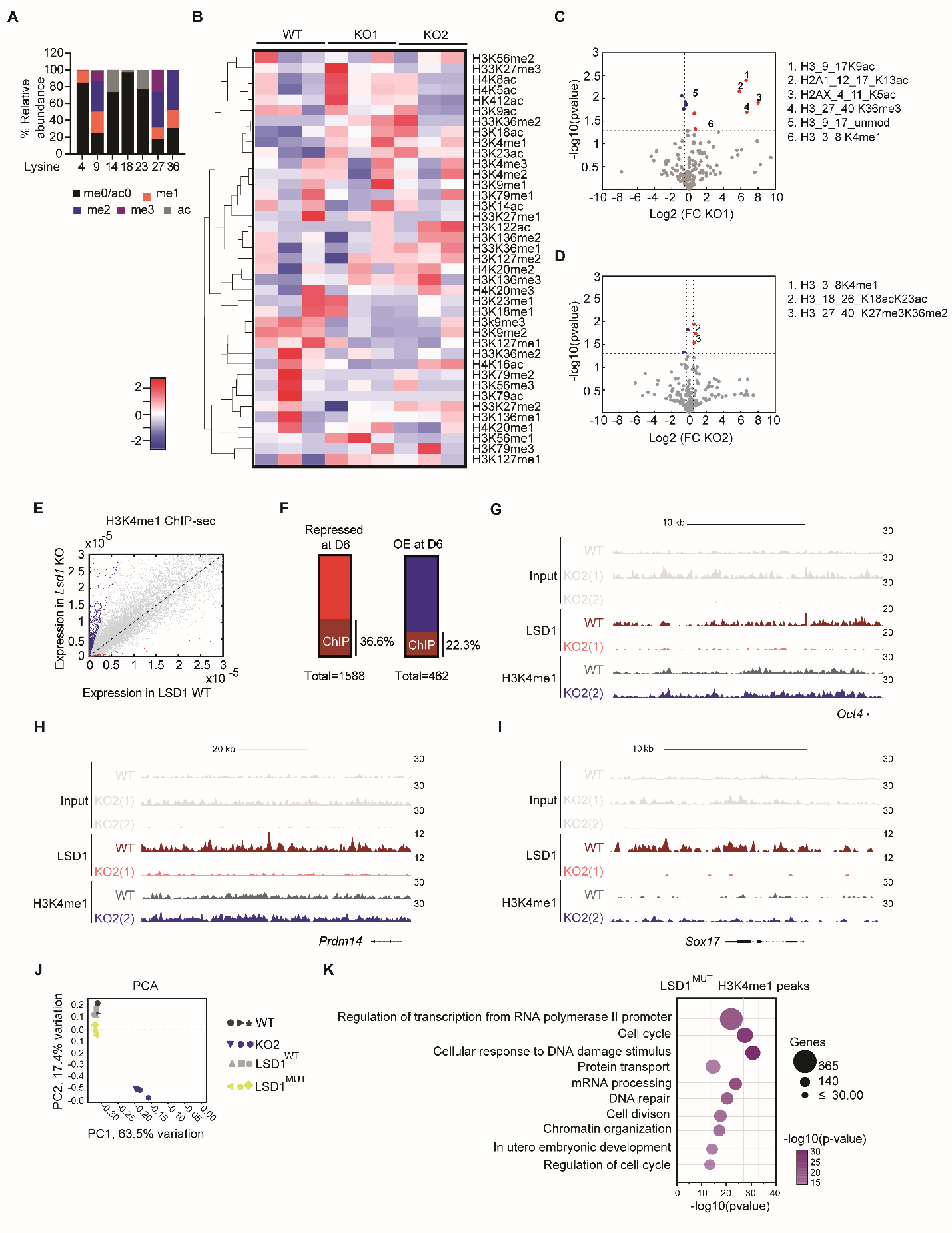
**

*Figure legends on the next page.*

**Supplementary Fig. 5: Genetic deletion of LSD1 affects H3K4me1 and H3K9me2/3, related to Fig. 3**

(A) Relative abundance of histone marks in different lysine positions on the N-terminal tail of histone H3 in WT mouse ESCs. The colour coding represents different post-translational modifications (PTM), and the y-axis represents their relative abundance.

(B) Heatmap of histone marks in WT, *Lsd1* KO1 and *Lsd1* KO2 mouse ESCs.

(C and D) Volcano plots showing the top enriched histone marks in (C) *Lsd1* KO1 and (D) KO2 relative to WT mouse ESCs. The significantly upregulated and downregulated are represented in red and blue respectively (p < 0.05 and FC > 1.5).

(E) Overlap of *Lsd1* RNA-seq with H3K4me1 ChIP-seq in WT and *Lsd1* KO2 mouse ESCs.

(F) Overlap of LSD1 ChIP-seq with publicly available RNA-seq data on RA-directed differentiation on indicated time points.

(G-I) LSD1 ChIP-seq signal and H3K4me1 ChIP-seq signal in WT and *Lsd1* KO2 mouse ESCs at the (G) *Oct4,* (H) *Prdm14 and* (I) *Sox17* enhancers. Respective inputs are depicted in grey.

(J) Principal component analysis of CUT&RUN LoV-U anti-H3K4me showing replicate clustering based on cell types (WT, *Lsd1* KO2, LSD1^WT^, and LSD1^MUT^ mouse ESCs).

(K) GO analysis of biological processes of genes associated with H3K4me1 peaks retrieved from CUT and RUN LoV-U exclusively in LSD1^MUT^ mouse ESCs.

**Supplementary Fig. 6:**

**
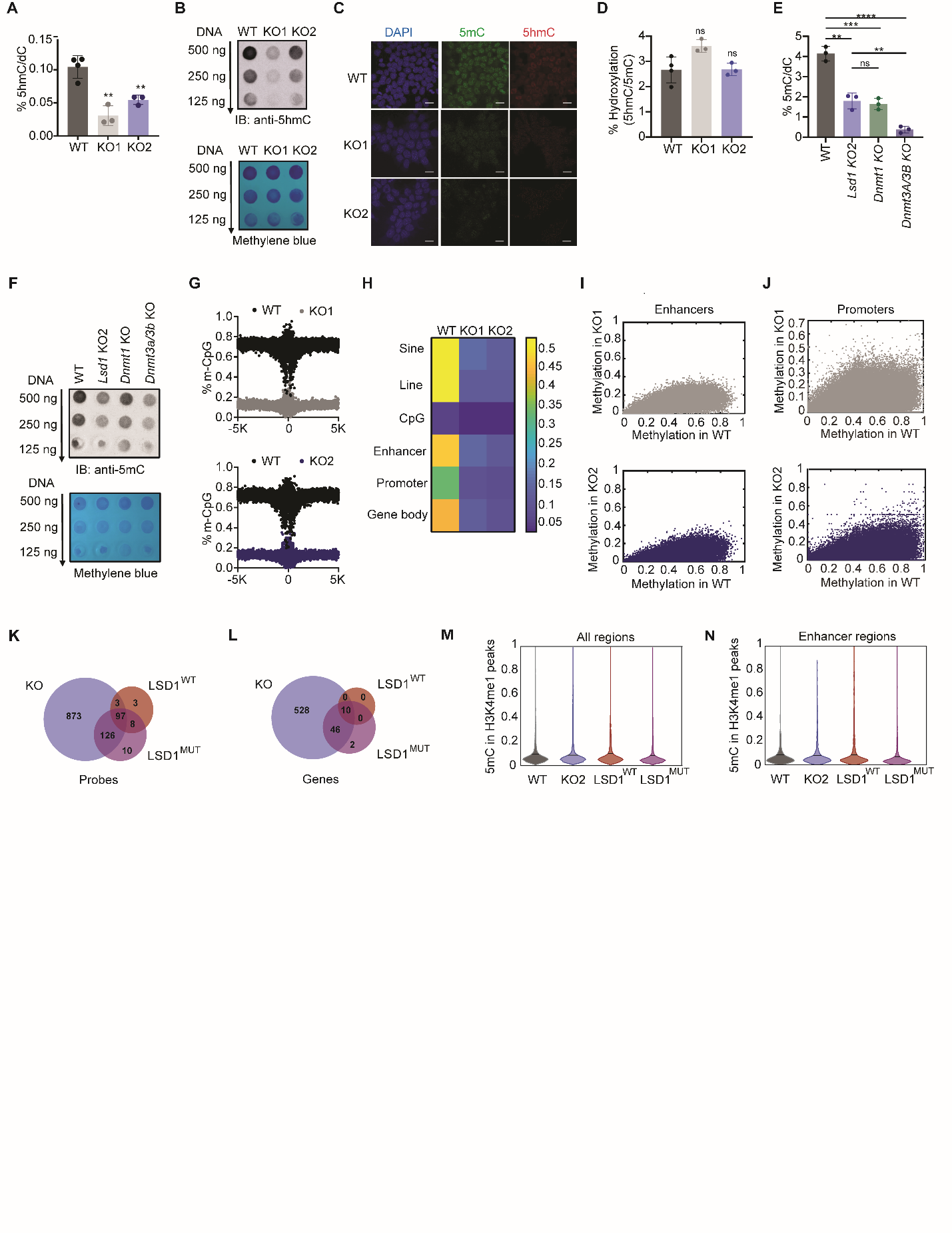
**

*Figure legends on the next page.*

**Supplementary Fig. 6: Loss of LSD1 affects global DNA methylation, related to Fig. 4**

(A and B) (A) LC-MS/MS quantification (B) DNA dot blot (left panel) of 5hmC on genomic DNA in WT, *Lsd1* KO1 and *Lsd1* KO2 mouse ESCs. 5hmC was normalized against 5mdC. Methylene blue staining was used as the loading control (right panel).

(C) Immunofluorescence of 5mC and 5hmC in WT, *Lsd1* KO1 and *Lsd1* KO2 mouse ESCs. DAPI was used as the nuclear marker. Scale bar, 20 μm.

(D) Percentage hydroxylation of cysteine in the genomic DNA extracted from WT, *Lsd1* KO1 and *Lsd1* KO2 mouse ESCs.

(E and F) (E) LC-MS/MS quantification and (F) DNA dot blot analysis of 5mC in genomic DNA of WT, *Lsd1* KO2, *Dnmt1* KO, and *Dnmt3a/3b* KO mouse ESCs (top panel). Methylene blue staining was used as the loading control (bottom panel).

(G) Composite plot of methylation in *Lsd1* KO1 (top panel) and *Lsd1* KO2 (bottom panel) compared to WT mouse ESCs across the CpG islands.

(H) Heatmap depicting the DNA methylation distribution in the different regulatory regions in WT, *Lsd1* KO1 and *Lsd1* KO2 mouse ESCs.

(I) Scatter plot of correlation analysis of enhancer methylation in *Lsd1* KO1 (top panel) and *Lsd1* KO2 (bottom panel) compared to WT mouse ESCs.

(J) Scatter plot of correlation analysis of promoter methylation in *Lsd1* KO1 (top panel) and *Lsd1* KO2 (bottom panel) compared to WT mouse ESCs.

(K and L) Venn diagram showing overlap of differentially methylated (K) probes (L) genes in *Lsd1* KO2, LSD1^WT^ and LSD1^MUT^ mouse ESCs.

(M and N) Violin plots for the overall distribution of DNA methylation in H3K4me1 peaks retrieved from CUT&RUN datasets in (M) all regions, (N) enhancer regions in WT, *Lsd1* KO2, LSD1^WT^, and LSD1^MUT^ mouse ESCs.

Statistical analysis: Two-tailed unpaired t-test (A, D, and E). ∗∗p < 0.01, ∗∗∗p < 0.001, ∗∗∗∗p < 0.0001 and ns- non- significant. Error bars denote mean ± SD. Each dot in the bar graphs represents independent biological replicates; n ≥ 3 (A, D, and E). Results are one representative of n = 3 independent experiments (B, C, and F).

**Supplementary Fig. 7:**

**
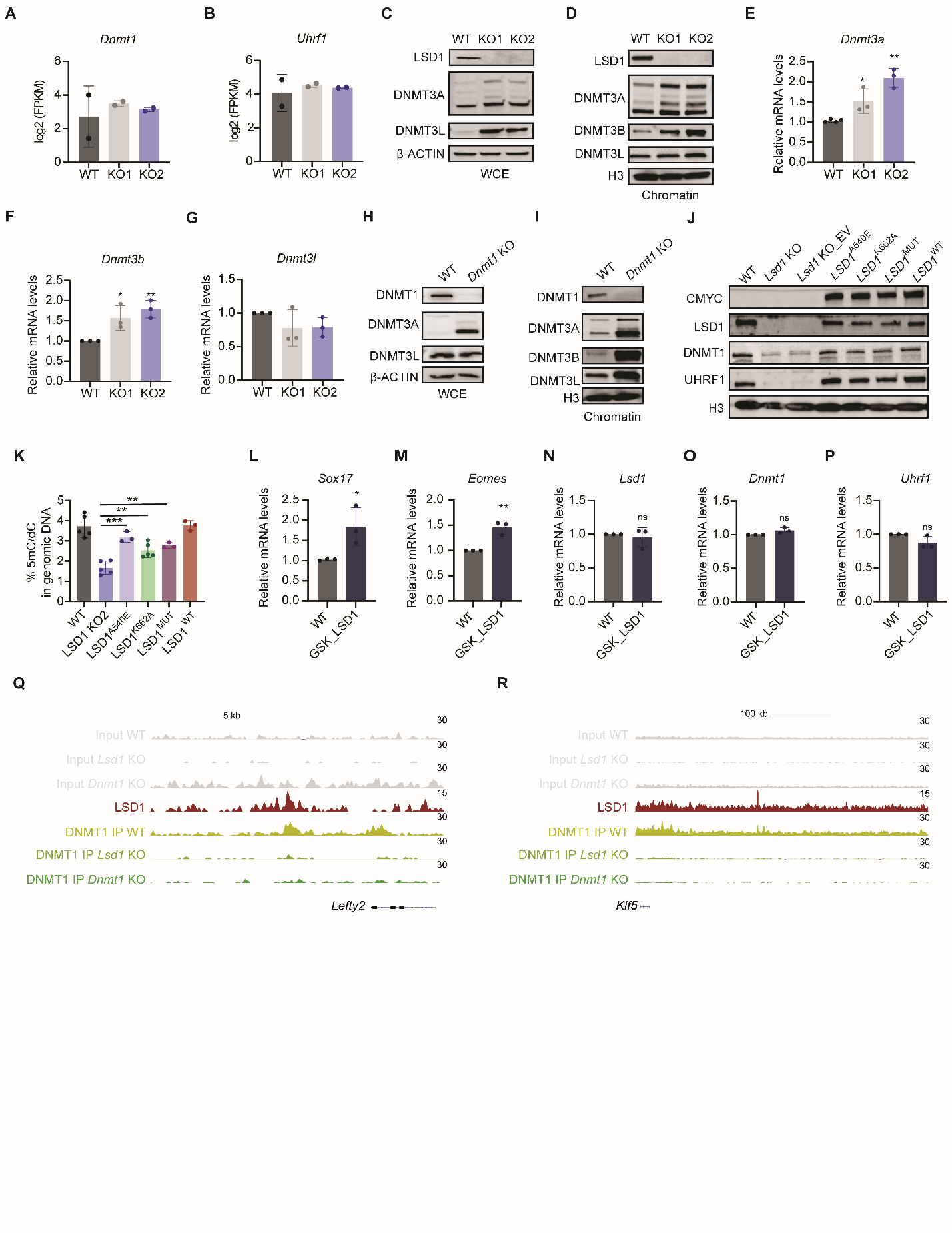
**

*Figure legends on the next page.*

**Supplementary Fig.7: LSD1 regulates DNMT1 and UHRF1 protein levels, related to Fig. 6**

(A and B) Expression level (FPKM) of *Dnmt1* and *Uhrf1* in WT, *Lsd1* KO1, and *Lsd1* KO2 mouse ESCs as determined by RNA-seq.

(C and D) Western blots of LSD1, DNMT3A, DNMT3L on the (C) WCE and (D) LSD1, DNMT3A, DNMT3B, and DNMT3L on the chromatin fractions of WT and *Lsd1* KO mouse ESCs. β-ACTIN and H3 are used as the loading controls.

(E-G) RT-qPCR of (E) *Dnmt3a*, (F) *Dnmt3b,* and (G) *Dnmt3l* in WT, *Lsd1* KO1, and *Lsd1* KO2 mouse ESCs. The mRNA levels are relative to the expression of WT.

(H and I) Western blots of DNMT1, DNMT3A, and DNMT3L on the (H) WCE and (I) LSD1, DNMT3A, DNMT3B, and DNMT3L on the chromatin fractions of WT and *Dnmt1* KO mouse ESCs. β-ACTIN and H3 are used as the loading controls.

1. Western blots of CMYC, LSD1, DNMT1, UHRF1 on the chromatin fractions of WT, *Lsd1* KO, *Lsd1* KO_EV, LSD1^A540E^_,_ LSD1^K622A^ ESCs, LSD1^WT^_,_ and LSD1^MUT^ mouse ESCs. H3 is used as the loading control.
2. LC-MS/MS quantification and of 5mC in genomic DNA of WT, *Lsd1* KO, LSD1^A540E^_,_ LSD1^K622A^, LSD1^WT^_,_ and LSD1^MUT^ mouse ESCs.

(L-P) RT-qPCR of (L) *Sox17,* (M) *Eomes* (N) *Lsd1* (O) *Dnmt1* and (P) *Uhrf1* in untreated (vehicle) and inhibitor (GSK_LSD1) treated WT mouse ESCs. The mRNA levels are relative to the expression of WT.

(Q and R) LSD1 ChIP-seq signal in WT mouse ESCs and DNMT1 ChIP-seq signal in WT, *Lsd1* KO2 and *Dnmt1* KO mouse ESCs at the (Q) *Lefty2,* and (R) *Klf5* enhancers. Respective inputs are depicted in grey.

Statistical analysis: Two-tailed unpaired t-test (E-G, and K-P). *p<0.05, ∗∗p < 0.01, ∗∗∗p < 0.001 and ns- non-significant. Error bars denote mean ± SD. Each dot in the bar graphs represents independent biological replicates; n =3 (E-G, K, and L-P). Results are one representative of n = 3 independent biological experiments (C, D, H, I, and J). Uncropped blots are represented in the source data.

**Supplementary Fig. 8:**

**
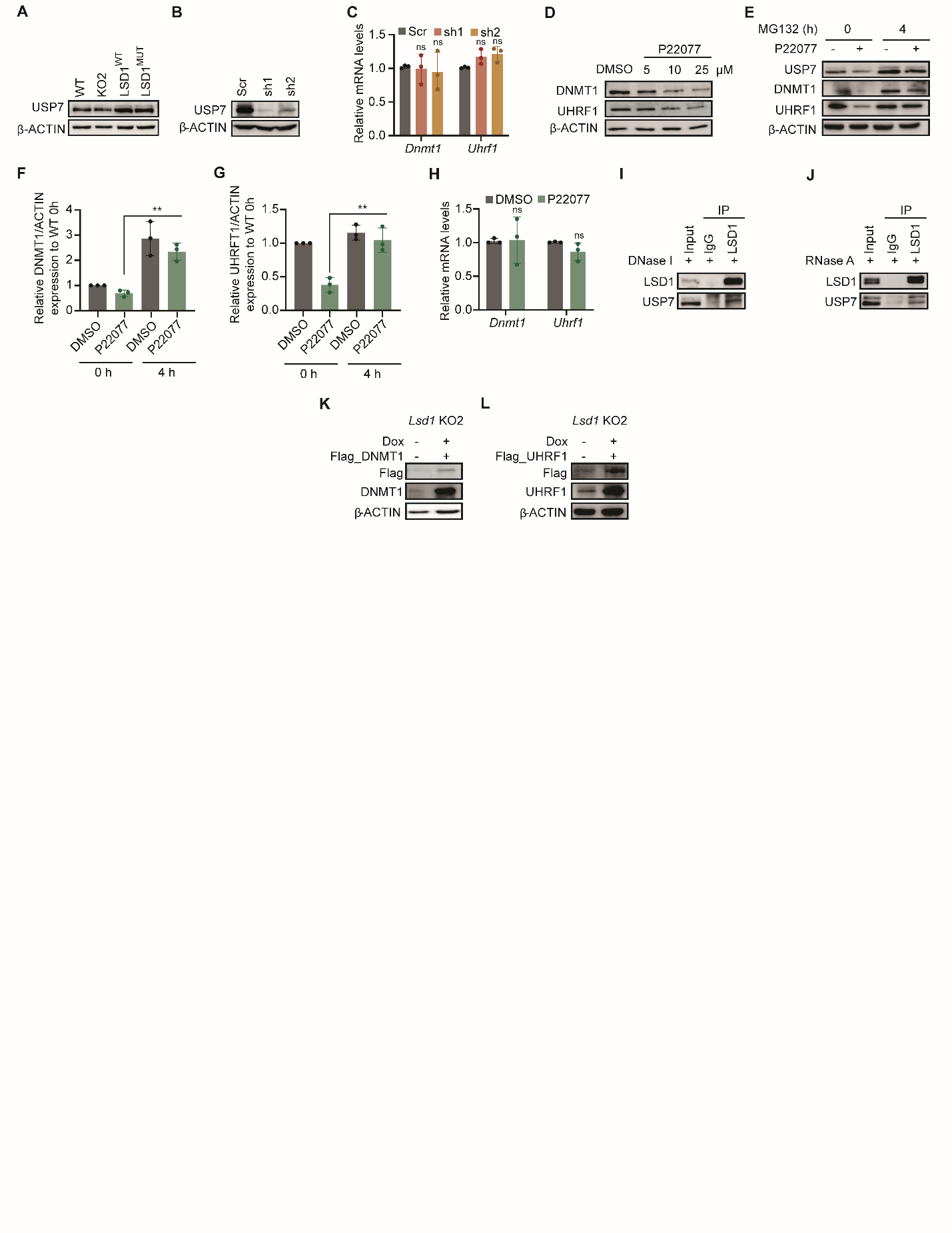
**

**Supplementary Fig. 8: Depletion of *Usp7* resulted in decreased DNMT1 and UHRF1, related to Fig. 7**

1. Western blot of USP7 on the WCE of WT, *Lsd1* KO2, LSD1^WT,^ and LSD1^MUT^ mouse ESCs. β-ACTIN is used as the loading control.
2. Western blot of USP7 on the WCE of WT ESCs upon scramble and *Usp7* knockdown. β-ACTIN is used as the loading control.
3. Bar graph depicting RT-qPCR analysis of *Dnmt1* and *Uhrf1* mRNA in the scramble and *Usp7* knockdown mouse ESCs. The mRNA levels are relative to the expression of scramble mouse ESCs.
4. Western blotting assay with antibodies against DNMT1 and UHRF1 on the WCE of DMSO and P22077 treated WT mouse ESCs. β-ACTIN is used as the loading control.
5. Western blot of USP7, DNMT1, and UHRF1 on the WCE of DMSO and P22077 treated WT mouse ESCs at indicated time points after MG132 treatment. β-ACTIN is used as the loading control.

(F and G) Quantification of relative (F) DNMT1 and (G) UHRF1 protein levels of western blot from (E) in indicated time points in DMSO and P22077 treated WT mouse ESCs.

(H) RT-qPCR analysis of *Dnmt1* and *Uhrf1* DMSO and P22077 treated WT mouse ESCs. The mRNA expressions are relative to DMSO WT ESCs.

(I and J) LSD1 immunoprecipitation in the presence of the (I) DNase I in nuclear fraction and (J) RNase A on the WCE of WT mouse ESCs followed by immunoblotting of USP7. The percentage of input used is 10%.

(K and L) Western blots of (K) Flag and DNMT1 in DNMT1-inducible *Lsd1* KO2 mouse ESCs and (L) Flag and UHRF1 in UHRF1-inducible *Lsd1* KO2 mouse ESCs in the presence (+) or absence (-) of doxycycline. β-ACTIN is used as the loading control.

Statistical analysis: Two-tailed unpaired t-test (C and F-H). ns- non-significant, ∗∗p < 0.01. Error bars denote mean ± SD. Each dot in the bar graphs represents independent biological replicates; n = 3 (C and F-H). Results are one representative of n = 3 independent biological experiments (A, B, D, E, I, J, K, and L). Uncropped blots are represented in the source data.

**Supplementary Fig. 9:**

**
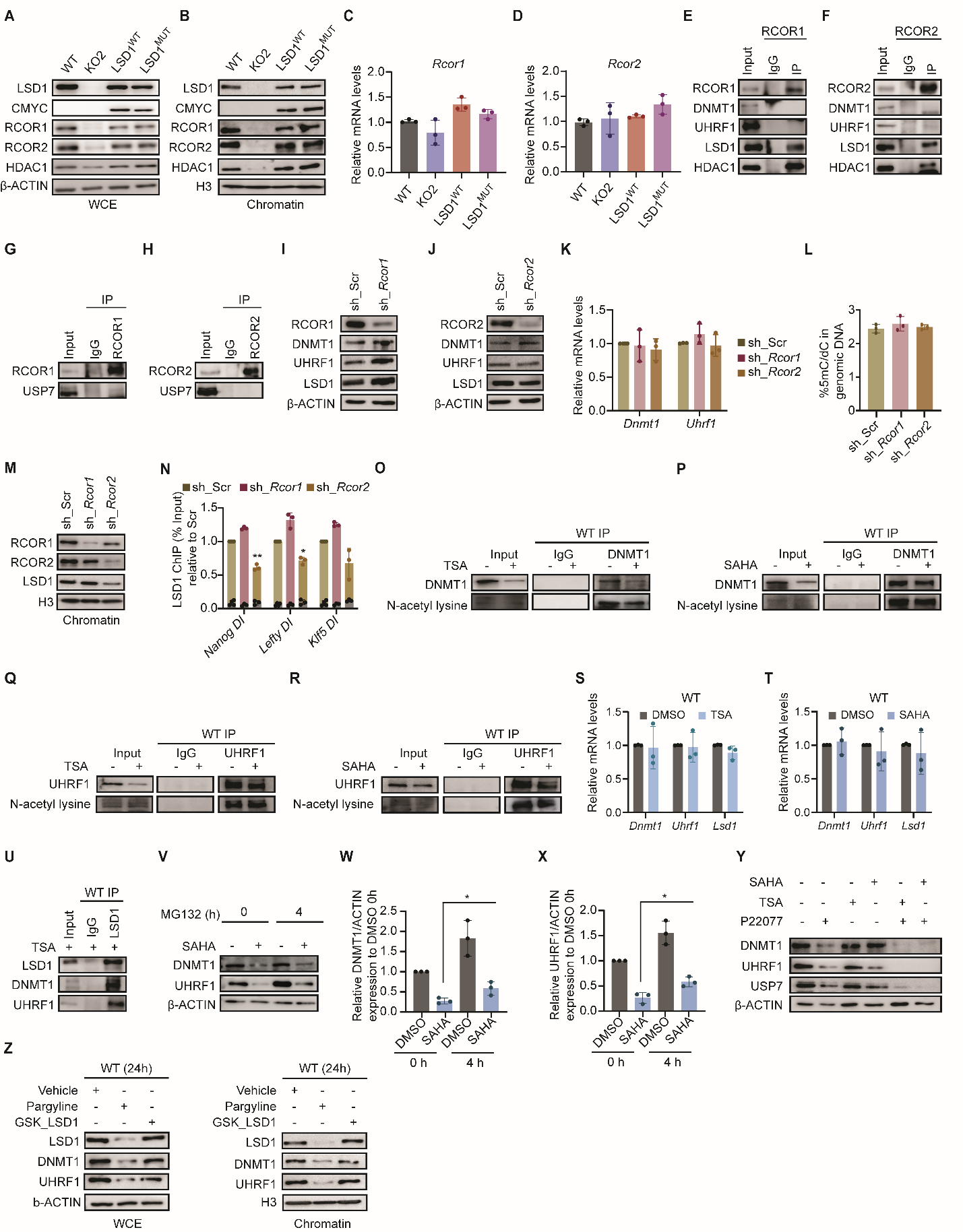
**

*Figure legends on the next page.*

**Supplementary Fig. 9: Deletion of LSD1 resulted in decreased DNMT1 and UHRF1 stability independent of RCOR1 and RCOR2 stability, related to Fig. 7**

(A and B) Western blots of LSD1, CMYC, RCOR1, RCOR2, and HDAC1 on the (A) WCE and (B) chromatin fractions of WT, *Lsd1* KO ESCs, LSD1^WT^_,_ and LSD1^MUT^ mouse ESCs. β-ACTIN and H3 are used as the loading controls.

(C and D) RT-qPCR of (C) *Rcor1* and (D) *Rcor2* in WT, *Lsd1* KO, LSD1^WT^_,_ and LSD1^MUT^ mouse ESCs. The mRNA levels are relative to the expression of WT.

(E and F) (E) RCOR1 and (F) RCOR2 immunoprecipitation on the WCE of WT mouse ESCs followed by DNMT1, UHRF1, LSD1, and HDAC1 immunoblotting. The percentage of input used is 10%.

(G and H) (G) RCOR1 and (H) RCOR2 immunoprecipitation on the WCE of WT mouse ESCs followed by immunoblotting of USP7. The percentage of input used is 10%.

(I) Western blots of RCOR1, DNMT1, UHRF1, and LSD1 in the scramble and sh_*Rcor1* mouse ESCs*.* β-ACTIN is used as the loading control.

1. Western blots of RCOR2, DNMT1, UHRF1, and LSD1 in the scramble and sh_*Rcor2* mouse ESCs*.* β-ACTIN is used as the loading control.
2. RT-qPCR analysis of *Dnmt1* and *Uhrf1* in the scramble, sh_*Rcor*1, and sh_*Rcor2* mouse ESCs*.* The mRNA levels are relative to the expression of a scramble.
3. LC-MS/MS quantification of 5mC in genomic DNA of scramble, sh_*Rcor*1, and sh_*Rcor2* mouse ESCs.
4. Western blots of RCOR1, RCOR2, and LSD1 on the chromatin fractions of scramble, sh_*Rcor*1, and sh_*Rcor2* mouse ESCs. H3 is used as the loading control.
5. ChIP-qPCR analysis of LSD1 enrichment at *Nanog,* *Lefty2, and Klf5* distal intergenic (DI) loci in the scramble, sh_*Rcor*1, and sh_*Rcor2* mouse ESCs. The data were normalized to input and represented relative to respective WT*.* IgG immunoprecipitation is depicted in black.

(O and P) DNMT1 immunoprecipitation on the WCE of (O) DMSO and TSA-treated WT mouse ESCs and (P) DMSO and SAHA-treated WT mouse ESCs followed by immunoblotting of N-acetyl lysine. The percentage of input used is 10%.

(Q and R) Immunoprecipitation of UHRF1 on the WCE of (Q) DMSO and TSA-treated WT mouse ESCs and (R) DMSO and SAHA-treated WT mouse ESCs followed by N-acetyl lysine immunoblotting. The percentage of input used is 10%.

(S and T) Bar graph representing RT-qPCR analysis of *Dnmt1*, *Uhrf1,* and *Lsd1* in (S) DMSO and TSA-treated WT mouse ESCs (T) DMSO and SAHA-treated WT mouse ESCs. The mRNA levels are relative to the expression of WT DMSO.

(U) LSD1 immunoprecipitation in WCE of WT mouse ESCs in the presence of TSA followed by DNMT1, UHRF1, and LSD1 immunoblotting. The percentage of input used is 10%.

(V) Western blotting of DNMT1 and UHRF1 on the WCE of DMSO and SAHA-treated mouse ESCs at the indicated time points of MG-132 treatment. β-ACTIN is used as the loading control.

(W and X) Protein recovery of (W) DNMT1 and (X) UHRF1 after quantification and normalization of the bands from (S9V). Protein expression is relative to DMSO WT mouse ESCs at 0 h.

(Y) Western blotting analysis of DNMT1, UHRF1, and USP7 in the presence of P22077, TSA, and SAHA on the WCE of WT mouse ESCs. β-ACTIN is used as the loading control.

(Z) Western blot of LSD1, DNMT1 and UHRF1 on the WCE (left panel) and chromatin (right panel) on pargyline and GSK_LSD1 treated WT mouse ESCs at indicated time point. β-ACTIN and H3 are used as the loading control.

Statistical analysis: Two-tailed unpaired t-test (C, D, K, L, N, S, and T). ns- nonsignificant (C, D, K, L, S and T) , *p<0.05, ∗∗p < 0.01. Error bars denote mean ± SD. Each dot in the bar graphs represents independent biological replicates; n =3 (C, D, K, L, N, S, and T). Results are one representative of n = 3 independent biological experiments (A, B, E, F, G, H, I, J, O, P, Q, R, U, V and Z). Uncropped blots are represented in the source data.
