# Supplementary material for: The scaffolding function of LSD1 controls DNA methylation in mouse ESCs
Source: Nat Commun. 2024 Sep 5;15:7758. doi: 10.1038/s41467-024-51966-7 (PMC11377572; doi:10.1038/s41467-024-51966-7)
Supplement: Supplementary file 3 — Description of Additional Supplementary Files [file 41467_2024_51966_MOESM3_ESM.pdf]

Description of Additional Supplementary Files

File Name: Supplementary Data 1

Description: RNA-seq in WT, *Lsd1* KO1 and *Lsd1* KO2 mouse ESCs and RNA-seq in WT, *Lsd1* KO2, LSD1^{WT} and LSD1^{MUT} mouse ESCs.

File Name: Supplementary Data 2

Description: LC-MS/MS of histone modifications in WT, *Lsd1* KO1 and *Lsd1* KO2 mouse ESCs.

File Name: Supplementary Data 3

Description: LSD1 ChIP-seq in WT mouse ESCs.

File Name: Supplementary Data 4

Description: H3K4me1 ChIP-seq in WT and *Lsd1* KO2 mouse ESCs.

File Name: Supplementary Data 5

Description: H3K4me1 peaks in WT, KO2, LSD1^{WT} and LSD1^{MUT} mouse ESCs retrieved from CUT&RUN method.

File Name: Supplementary Data 6

Description: Differentially methylated positions (DMPs) obtained from the Mouse Methylation MM285 BeadChIP microarray in *Lsd1* KO2, LSD1^{WT} and LSD1^{MUT} mouse ESCs compared to WT.

File Name: Supplementary Data 7

Description: RNA-seq and DMPs correlation analysis between WT and *Lsd1* KO2 mouse ESCs.

File Name: Supplementary Data 8

Description: DNMT1 ChIP-seq in WT and *Lsd1* KO2 mouse ESCs.

File Name: Supplementary Data 9

Description: Total proteomics analysis in *Lsd1* KO2, LSD1^{WT} and LSD1^{MUT} mouse ESCs compared to WT mouse ESCs.

File Name: Supplementary Data 10

Description: RNA-seq and total proteomics correlation analysis in KO2, LSD1^{WT} and LSD1^{MUT} mouse ESCs compared to WT mouse ESCs.

File Name: Supplementary Data 11

Description: Sequence of primers used in this study.
