# Supplementary material for: The scaffolding function of LSD1 controls DNA methylation in mouse ESCs
Source: Nat Commun. 2024 Sep 5;15:7758. doi: 10.1038/s41467-024-51966-7 (PMC11377572; doi:10.1038/s41467-024-51966-7)
Supplement: Supplementary file 15 — Reporting Summary [file 41467_2024_51966_MOESM15_ESM.pdf]

nature portfolio

Corresponding author(s): Francesca Aguilo

Last updated by author(s): 25/06/2024

Reporting Summary

Nature Portfolio wishes to improve the reproducibility of the work that we publish. This form provides structure for consistency and transparency in reporting. For further information on Nature Portfolio policies, see our Editorial Policies and the Editorial Policy Checklist.

Please do not complete any field with "not applicable" or n/a. Refer to the help text for what text to use if an item is not relevant to your study.
For final submission: please carefully check your responses for accuracy; you will not be able to make changes later.

Statistics

For all statistical analyses, confirm that the following items are present in the figure legend, table legend, main text, or Methods section.

n/a	Confirmed	
☐	☑	The exact sample size (*n*) for each experimental group/condition, given as a discrete number and unit of measurement
☐	☑	A statement on whether measurements were taken from distinct samples or whether the same sample was measured repeatedly
☐	☑	The statistical test(s) used AND whether they are one- or two-sided<br>*Only common tests should be described solely by name; describe more complex techniques in the Methods section.*
☒	☐	A description of all covariates tested
☐	☑	A description of any assumptions or corrections, such as tests of normality and adjustment for multiple comparisons
☐	☑	A full description of the statistical parameters including central tendency (e.g. means) or other basic estimates (e.g. regression coefficient) AND variation (e.g. standard deviation) or associated estimates of uncertainty (e.g. confidence intervals)
☐	☑	For null hypothesis testing, the test statistic (e.g. *F*, *t*, *r*) with confidence intervals, effect sizes, degrees of freedom and *P* value noted<br>*Give P values as exact values whenever suitable.*
☒	☐	For Bayesian analysis, information on the choice of priors and Markov chain Monte Carlo settings
☒	☐	For hierarchical and complex designs, identification of the appropriate level for tests and full reporting of outcomes
☒	☐	Estimates of effect sizes (e.g. Cohen's *d*, Pearson's *r*), indicating how they were calculated

Our web collection on statistics for biologists contains articles on many of the points above.

Software and code

Policy information about availability of computer code

Data collection	Carl Zen 2.3, Image Lab 6.0, Amersham Imager 680, CFX Maestro 2.3, Imagej, Illumina Nextseq 500/550, Illumina HiseqTM, QuantstudioTM,
Data analysis	BWA-meth algorithm, MethylDackel, SeSAme 1.14.2, R 4.0.3, pOOBAH algorithm, PCAtools 2.8.0, gplots 3.1.3, minifi 1.42.0, EpiProfile 2, bbmap bbduk (38.18), bowtie 2.4.5, Samtools 1.11, bedtools 2.23.0, SECAR 1.3, Deeptools 3.5.1-0, GREAT 4.0.4, HOMER 4.11, vsn 3.70.0, limma 3.58.1

For manuscripts utilizing custom algorithms or software that are central to the research but not yet described in published literature, software must be made available to editors and reviewers. We strongly encourage code deposition in a community repository (e.g. GitHub). See the Nature Portfolio guidelines for submitting code & software for further information.

Data

Policy information about availability of data

All manuscripts must include a data availability statement. This statement should provide the following information, where applicable:

- Accession codes, unique identifiers, or web links for publicly available datasets
- A description of any restrictions on data availability
- For clinical datasets or third party data, please ensure that the statement adheres to our policy

All next-generation sequencing data can be publicly accessed in ArrayExpress webserver (E-MTAB-14221).
By now, all next-generation sequencing data has been uploaded in https://figshare.com/s/fc83af7bfb83ef37b5d7
The mass spectrometry proteomics data have been deposited to the ProteomeXchange Consortium via the PRIDE partner repository with the dataset identifier PXD042495.

Research involving human participants, their data, or biological material

Policy information about studies with human participants or human data. See also policy information about sex, gender (identity/presentation), and sexual orientation and race, ethnicity and racism.

Reporting on sex and gender	Not relevant to the study
Reporting on race, ethnicity, or other socially relevant groupings	Not relevant to the study
Population characteristics	Not relevant to the study
Recruitment	Not relevant to the study
Ethics oversight	

Note that full information on the approval of the study protocol must also be provided in the manuscript.

Field-specific reporting

Please select the one below that is the best fit for your research. If you are not sure, read the appropriate sections before making your selection.

☑ Life sciences ☐ Behavioural & social sciences ☐ Ecological, evolutionary & environmental sciences

For a reference copy of the document with all sections, see nature.com/documents/nr-reporting-summary-flat.pdf

Life sciences study design

All studies must disclose on these points even when the disclosure is negative.

Sample size	The number biological replicates in each experiment were three or more than three except for figure 1P-S where n is 2 .<br>Each dot in the bar graphs represents independent biological replicates. For the line graphs, the number of replicates is mentioned in the source data.
Data exclusions	No data points were excluded.
Replication	The experiments were repeated at least three unless stated in the figure legends.
Randomization	Experiments were not randomized
Blinding	Alkaline phosphastase quantifications were performed blindly.

Behavioural & social sciences study design

All studies must disclose on these points even when the disclosure is negative.

Study description	
Research sample	
Sampling strategy	
Data collection	
Timing	
Data exclusions	
Non-participation	
Randomization	

Ecological, evolutionary & environmental sciences study design

All studies must disclose on these points even when the disclosure is negative.

Study description

Research sample

Sampling strategy

Data collection

Timing and spatial scale

Data exclusions

Reproducibility

Randomization

Blinding

Did the study involve field work? ☐ Yes ☐ No

Field work, collection and transport

Field conditions

Location

Access & import/export

Disturbance

Reporting for specific materials, systems and methods

We require information from authors about some types of materials, experimental systems and methods used in many studies. Here, indicate whether each material, system or method listed is relevant to your study. If you are not sure if a list item applies to your research, read the appropriate section before selecting a response.

Materials & experimental systems

n/a	Involved in the study	
☐	☑	Antibodies
☐	☑	Eukaryotic cell lines
☒	☐	Palaeontology and archaeology
☒	☐	Animals and other organisms
☒	☐	Clinical data
☒	☐	Dual use research of concern
☒	☐	Plants

Methods

n/a	Involved in the study	
☐	☑	ChIP-seq
☒	☐	Flow cytometry
☒	☐	MRI-based neuroimaging

Antibodies

Antibodies used

anti-LSD1 (Abcam, ab17721, 1:2500), anti-DNMT1 (Abcam, ab188453, 1:2500), anti-UHRF1 (Invitrogen, PA5-29884, 1:2000), anti-DNMT3A (Abcam, ab188470, 1:2500), anti-DNMT3B (Abcam, ab79822, 1:2000), anti-DNMT3L (Abcam, ab194094, 1:2000), anti-OCT4 (Santa-Cruz, SC-8628, 1:1500), anti-MYC (Cell Signalling, 2276S, 1:4000), anti-βACTIN (Sigma, A54411:5000), anti-H3 (Abcam, ab8895, 1:8000), anti-USP7 (Invitrogen, PA5-34911, 1:2000), anti-RCOR1 (Novus biologicals, NBP3-16225, 1:1500), anti-RCOR2 (Proteintech, 23969-1-AP, 1:2000), and anti-HDAC1 (Abcam, ab19845, 1:2500), anti-H3K4me1 (Abcam, ab8895, 2ug), anti-DNMT1 (Abcam, ab19905, 10ug), anti-lysine acetyl (Abcam, 9814S) and anti-Rabbit IgG (Abcam, ab37415, 5ug), anti-5hmC (Active Motif, 39791, 1:4000), anti-5mC (Active Motif, 39649, 1:4000), anti-LSD1 (Abcam, ab129195, 1:500), anti-OCT4 (Santa-Cruz, SC-8628, 1:250), anti-SSEA1 (Thermo Scientific, MA5-17042, 1:250), anti-Rabbit (Thermo Scientific, A-11011, 1:1000), and anti-Mouse (Thermo Scientific, A11029, 1:1000).

Validation

Validation of each antibody was checked in their respective company catalog. All antibodies were validated in wild type mouse ESCs.

Eukaryotic cell lines

Policy information about cell lines and Sex and Gender in Research

Cell line source(s)	CCE (CVCL_C313 were derived from blastocyst of male mice(https://www.cellosaurus.org/CVCL_C313) . Lsd1 KO mESC were generated from WT mESCs using CRISPR/cas9).
Authentication	None of the cell lines were authenticated independently.
Mycoplasma contamination	Cell lines were rountinely checked for mycoplasm by performing PCR on the media of freshly passaged cell lines.
Commonly misidentified lines (See ICLAC register)	None

Palaeontology and Archaeology

Specimen provenance	Not relevant to study
Specimen deposition	
Dating methods	

☐ Tick this box to confirm that the raw and calibrated dates are available in the paper or in Supplementary Information.

Ethics oversight	

Note that full information on the approval of the study protocol must also be provided in the manuscript.

Animals and other research organisms

Policy information about studies involving animals; ARRIVE guidelines recommended for reporting animal research, and Sex and Gender in Research

Laboratory animals	Not relevant to study.
Wild animals	
Reporting on sex	
Field-collected samples	
Ethics oversight	

Note that full information on the approval of the study protocol must also be provided in the manuscript.

Clinical data

Policy information about clinical studies

All manuscripts should comply with the ICMJE guidelines for publication of clinical research and a completed CONSORT checklist must be included with all submissions.

Clinical trial registration	Not relevant to study.
Study protocol	
Data collection	
Outcomes	

Dual use research of concern

Policy information about dual use research of concern

Hazards

Could the accidental, deliberate or reckless misuse of agents or technologies generated in the work, or the application of information presented in the manuscript, pose a threat to:

No	Yes	
☒	☐	Public health
☒	☐	National security
☒	☐	Crops and/or livestock
☒	☐	Ecosystems
☒	☐	Any other significant area

Experiments of concern

Does the work involve any of these experiments of concern:

No	Yes	
☒	☐	Demonstrate how to render a vaccine ineffective
☒	☐	Confer resistance to therapeutically useful antibiotics or antiviral agents
☒	☐	Enhance the virulence of a pathogen or render a nonpathogen virulent
☒	☐	Increase transmissibility of a pathogen
☒	☐	Alter the host range of a pathogen
☒	☐	Enable evasion of diagnostic/detection modalities
☒	☐	Enable the weaponization of a biological agent or toxin
☒	☐	Any other potentially harmful combination of experiments and agents

Plants

Seed stocks	Not relevant to study.
Novel plant genotypes	
Authentication	

ChIP-seq

Data deposition

☑ Confirm that both raw and final processed data have been deposited in a public database such as GEO.

☑ Confirm that you have deposited or provided access to graph files (e.g. BED files) for the called peaks.

Data access links<br>*May remain private before publication.*	
Files in database submission	All sequencing data can be publicly accessed in ArrayExpress weberver (E-MTAB-14221).
Genome browser session<br>(e.g. UCSC)	

Methodology

Replicates	The number of biological replicates for each ChIP-sequencing is two.
Sequencing depth	Paired-end read sequencing (20M per sample).
Antibodies	anti_LSD1 (abcam, ab17721), anti-H3K4me1 (abcam, ab8895) and anti-DNMT1 (abcam, ab19905).
Peak calling parameters	Peak calling was performed using MACS2 with pooled IPs and inputs (FDR less than 0.05).
Data quality	Spike-in control (human MCF10A cell line) was used for normalization of the ChIP-seq reads.Raw reads were aligned to the human (hg38) and mouse (mm10) genomes using BWA. The final mouse BAM files were normalized using the number of reads that uniquely mapped to the human genome
Software	BWA 0.7.18, MACS2 2.2.9, chIPseekeR 1.28.3, bedtools 2.28, HOMER 4.11, MATLAB 2021b, GBiB July 2022, EaSEQ 1.2 and R 4.4.

Flow Cytometry

Plots

Confirm that:

- [ ] The axis labels state the marker and fluorochrome used (e.g. CD4-FITC).
- [ ] The axis scales are clearly visible. Include numbers along axes only for bottom left plot of group (a 'group' is an analysis of identical markers).
- [ ] All plots are contour plots with outliers or pseudocolor plots.
- [ ] A numerical value for number of cells or percentage (with statistics) is provided.

Methodology

Sample preparation

Instrument

Software

Cell population abundance

Gating strategy

- [ ] Tick this box to confirm that a figure exemplifying the gating strategy is provided in the Supplementary Information.

Magnetic resonance imaging

Experimental design

Design type

Design specifications

Behavioral performance measures

Imaging type(s)

Field strength

Sequence & imaging parameters

Area of acquisition

Diffusion MRI ☐ Used ☐ Not used

Preprocessing

Preprocessing software

Normalization

Normalization template

Noise and artifact removal

Volume censoring

Statistical modeling & inference

Model type and settings

Effect(s) tested

Specify type of analysis: ☐ Whole brain ☐ ROI-based ☐ Both

Statistic type for inference

(See Eklund et al. 2016)

Correction

Models & analysis

N

n/a	Involved in the study
☐	☐ Functional and/or effective connectivity
☐	☐ Graph analysis
☐	☐ Multivariate modeling or predictive analysis

Functional and/or effective connectivity

Graph analysis N/a

Multivariate modeling and predictive analysis

This checklist template is licensed under a Creative Commons Attribution 4.0 International License, which permits use, sharing, adaptation, distribution and reproduction in any medium or format, as long as you give appropriate credit to the original author(s) and the source, provide a link to the Creative Commons license, and indicate if changes were made. The images or other third party material in this article are included in the article's Creative Commons license, unless indicated otherwise in a credit line to the material. If material is not included in the article's Creative Commons license and your intended use is not permitted by statutory regulation or exceeds the permitted use, you will need to obtain permission directly from the copyright holder. To view a copy of this license, visit http://creativecommons.org/licenses/by/4.0/
